# Compositional Bias of Intrinsically Disordered Proteins and Regions and Their Predictions

**DOI:** 10.3390/biom12070888

**Published:** 2022-06-25

**Authors:** Bi Zhao, Lukasz Kurgan

**Affiliations:** Department of Computer Science, Virginia Commonwealth University, Richmond, VA 23284, USA

**Keywords:** intrinsic disorder, intrinsically disordered proteins, intrinsic disordered regions, disorder scale, disorder propensity, amino acids, amino acid bias, predictive performance, disorder prediction

## Abstract

Intrinsically disordered regions (IDRs) carry out many cellular functions and vary in length and placement in protein sequences. This diversity leads to variations in the underlying compositional biases, which were demonstrated for the short vs. long IDRs. We analyze compositional biases across four classes of disorder: fully disordered proteins; short IDRs; long IDRs; and binding IDRs. We identify three distinct biases: for the fully disordered proteins, the short IDRs and the long and binding IDRs combined. We also investigate compositional bias for putative disorder produced by leading disorder predictors and find that it is similar to the bias of the native disorder. Interestingly, the accuracy of disorder predictions across different methods is correlated with the correctness of the compositional bias of their predictions highlighting the importance of the compositional bias. The predictive quality is relatively low for the disorder classes with compositional bias that is the most different from the “generic” disorder bias, while being much higher for the classes with the most similar bias. We discover that different predictors perform best across different classes of disorder. This suggests that no single predictor is universally best and motivates the development of new architectures that combine models that target specific disorder classes.

## 1. Introduction

Intrinsically disordered regions (IDRs) are highly flexible segments in protein sequences that a lack well-defined tertiary structure and typically take form of conformational ensembles under physiological conditions [1,2,3,4]. Intrinsically disordered proteins (IDPs) include one or more IDRs. Recent bioinformatics studies have suggested that approximately a third of eukaryotic proteins have long IDRs that are composed of 30+ disordered amino acids (AAs) [5,6,7,8]. Sequences of IDRs have compositional biases, typically being enriched in charged and polar AAs and depleted in bulky hydrophobic residues [1,4,9,10,11,12,13,14]. To this end, the TOP-IDP scale was designed to quantify the intrinsic propensities of AAs for the disordered vs. structured conformations [15].

Several databases, including DisProt [16,17], PED [18,19], PDB [20,21], IDEAL [22], DIBS [23], FuzDB [24,25] and MFIB [26], provide access to the experimentally characterized IDPs and IDRs. However, they only cover a small fraction of these data, with approximately 2400 IDPs in DisProt and over 20,000 in PDB [16,27,28]. The observation that disorder is an inherent/intrinsic property of the AA sequences [1,9,10] motivates the development of accurate computational tools that predict disorder in a given protein sequence. These convenient and fast tools can be used to bridge the annotation gap and stimulate the rapid acceleration of research into IDPs and IDRs [29]. Over 100 disorder predictors have already been developed [30]. Many comprehensive studies summarize, survey and comparatively assess disorder predictors [28,30,31,32,33,34,35,36,37,38,39,40,41,42,43,44,45,46,47,48,49,50,51]. These include several community assessments, such as Critical Assessment of Structure Prediction (CASP) between CASP5 and CASP 10 [45,46,47,48,50,51], and more recently the Critical Assessment of Intrinsic Protein Disorder (CAID) [49]. These studies describe currently available tools, identify interesting trends in the development of new methods, provide practical advice on how to identify and use the best predictors, and point to future directions.

One interesting direction is to explore the underlying diversity of intrinsic disorder [52,53,54]. Studies show that IDRs are instrumental for a broad spectrum of cellular functions including molecular recognition, signaling, regulation, phase separation, translation, transcription, alternative splicing, protein–protein and protein–nucleic acids interactions [53,55,56,57,58,59,60,61,62,63,64,65,66,67,68,69,70], and some of them are multifunctional [71,72]. IDRs also vary in their conformational space and they are correspondingly categorized into the native coils, native pre-molten globules and native molten globules [3,73]. Moreover, they also differ in size and placement in the sequence. Short IDRs are often located at the termini of the protein sequence while very long IDRs can span the entire length of the protein sequence [3,54,74,75]. Moreover, short IDRs were observed to have different amino acid compositions when compared to long IDRs [76,77] and correspondingly, some predictors, such as the popular IUPred [78,79,80,81], predict them separately. The diversity of sizes, locations and functions of IDRs likely results in the presence of different biases in their corresponding sequences, which cannot be captured with a single overarching TOP-IDP scale.

To this end, we investigated the compositional bias of IDRs in the context of their size and a coarsely-defined function. Moreover, using the recently released CAID results, we investigated whether the putative disorder produced by leading disorder predictors is characterized by correspondingly different AA-level biases and whether these biases influence their predictive performance. Finally, we studied whether the predictor-level biases affect their ability to accurately identify different types of disorder defined by size and function. This leads to interesting observations that may inspire the development of novel and potentially more accurate disorder predictors.

## 2. Materials and Methods

### 2.1. Data

The recent CAID experiment provides a well-annotated and large benchmark dataset that was used to assess modern disorder predictors [49]. The authors of these predictors were excluded from the process of data collection, annotation and assessment. Moreover, the underlying data were collected after these methods were trained, ensuring that the results can be reliably used to assess and compare these predictors. We obtained the experimentally annotated CAID data, including annotations of IDRs and binding IDRs from https://idpcentral.org/caid/data/1/reference/disprot-disorder.txt (accessed on 22 December 2021) and https://idpcentral.org/caid/data/1/reference/disprot-binding.txt (accessed on 22 December 2021). This dataset includes 652 protein sequences with 337,908 residues, including 838 IDRs and 54,820 disordered residues, among which there are 256 disordered binding regions and 21,389 disordered binding residues. We summarize the details in Table 1. We used these data to investigate the AA-level biases of disorder and to categorize the disorder based on the size (short, long and fully disordered) and function (binding IDRs and non-binding IDRs). We also collected predictions generated by the top 10 of 32 disorder predictors that participated in the CAID assessment from https://idpcentral.org/caid/data/1/predictions/ (accessed on 17 January 2022). These predictors include (in alphabetical order): AUCpreD [82], AUCpreD-np [82], DisoMine [83], flDPlr [84], flDPnn [84], Predisorder [85], RawMSA [86], SPOT-Disorder1 [87], SPOT-Disorder2 [88] and SPOT-Disorder-Single [89]. We excluded the ESpritz-D method that is listed in the CAID experiment since this tool was authored by the organizers of CAID and it was not officially evaluated. These data allow us to study the compositional biases of the putative disordered residues identified by these methods and to investigate the relations of these biases with the corresponding predictive performance.

### 2.2. Categorization of IDRs

IDRs vary greatly in their length and function, which in our case, divides these regions into ligand binding and non-binding [54,61,62,64,65,74]. Our motivation for this coarse-grained categorization of function stems from the focus on this aspect of disorder in the recent CAID experiment [49], the high significance of the disorder-driven interactions in the context of cellular functions of disorder [61,62,64,65], and the fact that this is by far the most commonly annotated disorder function in the largest database of disorder functions annotations, DisProt [16,90].

We divided IDRs into four categories based on their length, the disordered content of the IDR-containing IDP and the annotation of binding. The disorder content is calculated as the total number of annotated disordered residues divided by the length of a given protein sequence. Using the annotations from CAID [49], which are in turn sourced from DisProt [16], IDRs are defined as the segments of at least ten consecutive disordered residues [16,91,92]. The first category are fully disordered proteins. The IDRs in this category cover at least 80% of a given IDP (disorder content ≥ 0.8). Approximately 10% of IDRs in our dataset belong to this category, including 57 regions and 9208 disordered residues. The second category are the short IDRs that include IDRs with ≥10 and <15 consecutive disordered residues that are in proteins with a disorder content < 0.3. Our dataset includes 148 short IDRs that consist of 1810 disordered residues. The third category are long IDRs that are over 70 residues in length and present in IDPs with the disorder content ranging between 0.3 and 0.8. There are 77 long IDRs with 14,935 disordered residues in our dataset. The fourth category is that of disordered binding regions. These overlap with the former three categories and their defining characteristic is that they interact with ligands. There are 256 disordered binding regions that are composed of 21,389 disordered binding residues in our dataset. While the breakdown by the region length might be seen as somehow arbitrary, we note that we did not attempt to rigorously define these categories but rather to identify large collections of IDRs that are diverse in length and cover a sufficient amount of data for performing a robust statistical analysis. We summarize these data in Table 1.

### 2.3. Computational Analysis

Composition Profiler is a popular web-based tool that can be used to investigate the differences of amino acid compositions between collections of proteins or protein regions [93]. We applied this tool to quantify the compositional biases of AAs in various collections of IDRs and across the entire CAID dataset by comparing them with a background sample, which consists of the non-disordered residues from the CAID dataset. We note that the background is the same, allowing us to compare these scales side by side. Moreover, we computed the composition biases of the disorder predictions by comparing the putative disordered residues against the background that consists of the putative non-disordered residues generated by the top ten disorder predictors from the CAID experiment. Altogether, this analysis produced 15 scales (CAID, fully disordered; short IDRs; long IDRs; binding IDRs; plus ten predictors) that quantify the propensity of AAs for the native and predicted disorder.

We investigated the correlations between these scales to quantify their similarity. We used the Kendall rank correlation coefficients (KCCs) that measure the similarity of the orderings of given scales when the values of each scale are ranked [94]. This is motivated by the observations that the scales cover both positive and negative values (i.e., positive when residues are enriched in IDRs vs. negative when enriched in ordered regions) and that the ranges of their values differ across scales.

We also quantified the statistical significance of the differences in the predictive performance of disorder predictions. Inspired by recent works [31,32,40,95], this test aims to assess the robustness of the differences to the use of different datasets of proteins, i.e., whether a given prediction is better than another prediction across diverse datasets. First, we randomly bootstrapped 50% of proteins from the CAID dataset 100 times, and computed the corresponding 100 assessments. We compared the corresponding 100 results using the Student *t*-test if the data were normal; otherwise, we used the non-parametric Wilcoxon rank test. We tested normality using the Anderson–Darling test at the *p*-value of 0.05.

## 3. Results and Discussion

### 3.1. Compositional Biases from the TOP-IDP Scale and the CAID Data Are Consistent

**Figure 1:**
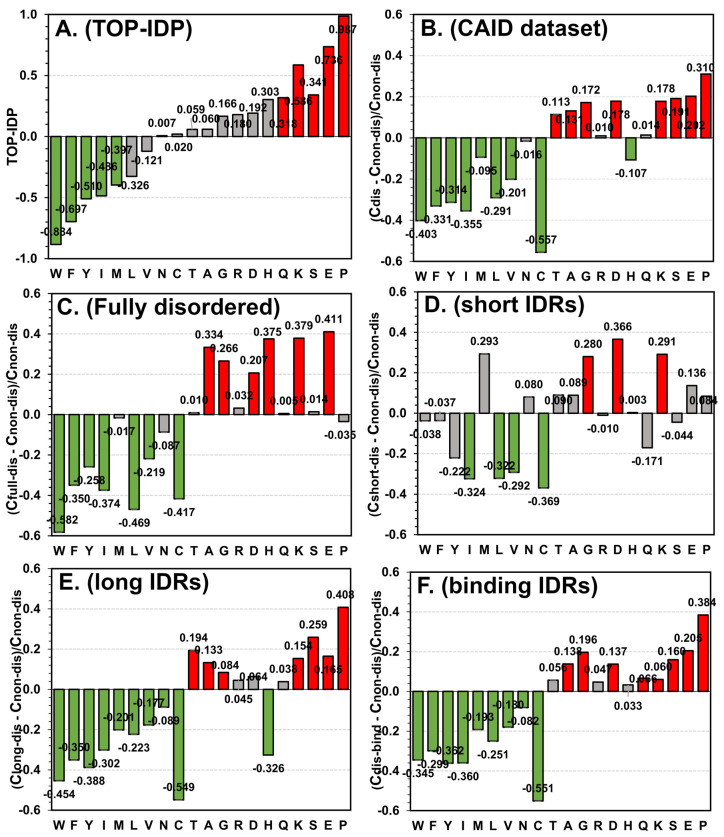
Compositional bias of intrinsic disorder measured for different collections of disordered proteins and regions. (**A**) TOP-IDP scale; (**B**) CAID dataset; (**C**) fully disordered proteins in CAID; (**D**) short IDRs in CAID; (**E**) long IDRs in CAID; and (**F**) disordered binding regions in CAID. The amino acids on the *x* axis are sorted according to the TOP-IDP scale in the way that is consistent with the original article (data for panel A was adapted from Ref. [15]), from the most order promoting to the most disorder promoting. The propensities are color-coded where green denotes statistically significant depletion; red denotes statistically significant enrichment; and gray denotes that the difference is not statistically significant at the *p*-value of 0.05. Values of the disorder propensities are shown at the top of the bars.

We computed and investigated the AA bias (i.e., disorder scale) for the disorder in the CAID dataset. The comparison of the published TOP-IDP scale (Figure 1A) and the new scale based on the CAID dataset (Figure 1B) reveals that they are similar. The KCC of the two scales is 0.691, which means that they are highly correlated. The five-order-promoting AAs (W, F, Y, I and M) and four-disorder-promoting AAs (P, E, S and K) in TOP-IDP concur with their designation in the CAID dataset scale. The CAID scale designates the statistically disorder-promoting Q from TOP-IDP as it was not significantly different but with a slight bias towards disorder. Several other statistically significant biases in the CAID scale that include enrichment in order for L and V and enrichment in disorder for T, A, G and D are also consistent with the direction of biases in the TOP-IDP scale. The two key differences are the significant enrichment in the structured conformations for C and H in the CAID scale where these AAs have positive and not statistically significant bias toward disorder in the TOP-IDP scale. Interestingly, the TOP-IDP analyses of the bias that relies on the experimental data from DisProt ranks the AAs according to the disorder propensity as follows: P (propensity of 1.0), E (0.78), S (0.71) Q (0.66), K (0.59), A (0.45), G (0.44), D (0.41), T (0.40), R (0.39), M (0.29), N (0.28), V (0.26), H (0.26), L (0.20), F (0.12), Y (0.11), I (0.09), W (0.00) and C (0.00) [12,13,14]. Another study that utilizes a different source of data, primarily depending on the protein structures from PDB, finds that IDRs are depleted in W, C, F, I, Y, V, L and N; enriched in A, R, G, Q, S, P, E and K; while H, M, T and D lack a significant bias [96]. Both of these findings are in close agreement with our results, including the observation that C and H are not enriched in IDRs. The biggest outlier, cysteine (C), is considered order-promoting due to the fact that this AA forms inter- or intramolecular disulfide bonds. However, some protein domains were shown to contain disordered regions interspersed with flanking cysteines, where cysteine-induced disulfide bridges promote disorder-to-order and order-to-disorder transitions [97]. This is possibly why the TOP-IDP scale records a different bias for this AA.

### 3.2. Compositional Biases Differ between Different Categories of IDRs

**Figure 2:**
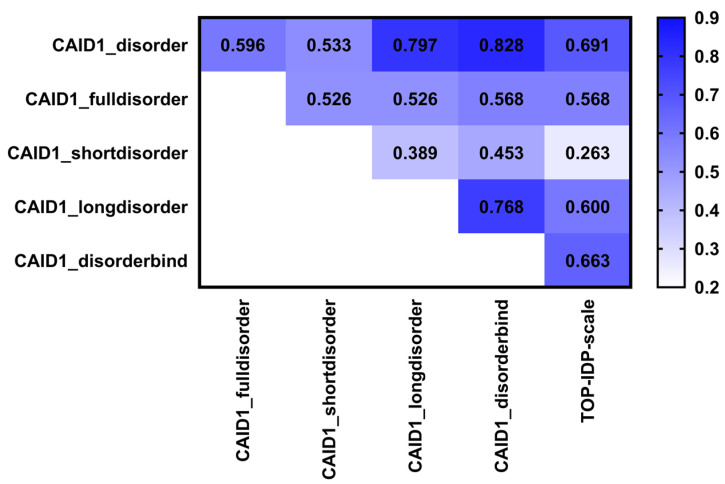
Kendall rank correlation coefficients (KCCs) between the AA biases for disorder in the overall CAID dataset, each of the four categories of IDRs (short, long, fully disordered and binding), and the TOP-IDP scale. The KCC values are color-coded from light blue for low values to dark blue for high values.

We compute and investigate the disorder scales for the fully disordered proteins (Figure 1C), the short IDRs (Figure 1D), the long IDRs (Figure 1E) and the binding disordered regions (Figure 1F). Figure 1 compares these four scales with the TOP-IDP scale (Figure 1A) and the disorder in the entire CAID dataset (Figure 1B). Figure 2 gives the complete set of KCCs for all the pairs of scales. The top row in Figure 2 focuses on the correlations between the four scales and the broad collection of disorder in CAID. We find that these KCC values range from a modest level at 0.533 for the short IDRs scale to a high value at 0.828 for the binding IDRs scale. Moreover, the two scales that are highly correlated with the CAID scale, for the long IDRs (KCC = 0.797) and the binding IDRs (KCC = 0.828), are also similar to one another (KCC = 0.768). This is regardless of the fact that the binding regions are much shorter than long IDRs (Table 1). In contrast, the two scales that have modest correlations with the CAID scale, for the short IDRs (KCC = 0.533) and the fully disordered proteins (KCC = 0.596), have a similarly modest correlation with each other (KCC = 0.526). Interestingly, the correlations of the short IDRs scale with the other three targeted scales (i.e., scales for the long IDRs, binding IDRs and fully disordered) range between 0.435 and 0.526, suggesting that this scale is rather unique/dissimilar to the other three scales. This result is supported by a past study that similarly found that the AA compositions are significantly different between short IDRs (<10 residues) and long IDRs (≥30 residues) [77]. Furthermore, we find that the fully disordered scale registers relatively low KCC values between 0.526 and 0.568 when compared with the other three targeted scales. We also find that the correlations of the four scales with the TOP-IDP scale follow the same pattern as their correlations with the CAID data scale (i.e., the KCC of the binding IDRs > KCC of the long IDRs > KCC of the fully disordered IDPs > KCC of the short IDRs), except that the KCC values are lower. The lower values stem from the differences between the TOP-IDP and CAID scales that we discussed in Section 3.1. These correlation-based observations also agree with a visual inspection of the raw data in Figure 1. Scales in Figure 1E,F are relatively similar, while the scales in Figure 1C,D are different from each other and the other two scales. One of the key differences that we observe is for proline, the residue with the highest propensity for disorder in our CAID-based scale and in several other studies [12,13,14,15]. We find that proline is significantly and highly enriched in the binding and long IDRs, while being neutral for the short IDRs and fully disordered proteins. High levels of proline in the disordered binding regions concur with observations in the literature [12,98]. Moreover, proline is suggested as a modulator of secondary structures of neighboring AAs [12,99], which might explain its enrichment in the long IDRs where there is a sufficient number of residues to form residual structural elements that could be modulated and formed upon disorder-to-order transitions. Taken together, this analysis reveals three distinct types of disorder biases: one that encompasses the long and binding IDRs; the second for short IDRs; and the third for the fully disordered proteins. We also note that our results are consistent with prior studies that similarly point to substantial differences between short and long IDRs [76,77].

### 3.3. Compositional Biases for the Putative and Native Disorder Are Highly Correlated and These Correlations Influence Predictive Performance

**Figure 3:**
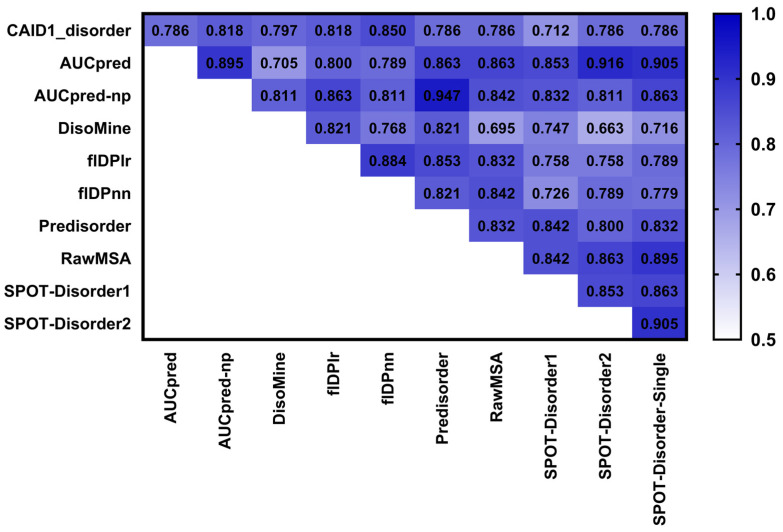
Kendall rank correlation coefficients (KCCs) between the AA biases for disorder in the overall CAID and putative disorder generated by the top ten predictors from the CAID experiment. The KCC values are color-coded from light blue for low values to dark blue for high values. Disorder predictors are sorted alphabetically.

We then investigate the compositional biases for the putative disorder generated by the top ten predictors evaluated in the CAID experiment. For reference, these methods secure areas under the ROC curve (AUC) values of 0.814 (flDPnn), 0.793 (flDPlr), 0.780 (RawMSA), 0.765 (DisoMine), 0.760 (SPOT-Disorder2), 0.757 (AUCpreD), 0.757 (SPOT-Disorder-Single), 0.751 (AUCpreD-np), 0.747 (Predisorder) and 0.744 (SPOT-Disorder1); and we reproduce these results from Figure 2 in the CAID article [49]. The top row in Figure 3 quantifies and compares the correlations between the CAID-based scale and the ten scales for the predicted disorder. We find that the putative disorder generated by the top ten predictors has a compositional bias that is very similar to the bias of the native disorder. The corresponding KCCs that are over 0.7 imply high correlations. This suggests that the ability of these methods to correctly predict disorder coincides with the accurate compositional bias of their predictions.

Furthermore, we find that the KCC values with the CAID-based scale range between 0.712 for SPOT-Disorder1, which is ranked 10th in CAID, and 0.850 for flDPnn, which is ranked 1st in CAID [100]. To this end, we further investigate whether these differences are correlated with the underlying predictive performance. The Pearson Correlation Coefficient (PCC) that quantifies the relation between the predictive performance measured with the AUC and corresponding KCC values of the ten predictors equals 0.703. This points to the strong effect that the level of agreement between the compositional biases of disorder predictions and the native disorder has on the performance of the best disorder predictors. This is an interesting observation since these methods utilize different training datasets, many distinctive types of inputs (e.g., protein sequences, evolutionary features, putative structural features, physicochemical properties of AAs) and various kinds of predictive models (e.g., support vector machines, decision trees, random forests, shallow and deep neural networks) [36,37,40,101]. However, the differences in their predictive performance can be largely explained by the quality of the compositional bias of the putative disorder that they generate.

Figure 3 also quantifies the correlations of the compositional biases of the putative disorder produced by different predictors. We find that these correlations vary widely between 0.663 (SPOT-Disorder2 with DisoMine) and 0.947 (Predisorder with AUCpred-np). This suggests that the predictions of different methods produce different biases, motivating an analysis that investigates whether their predictive performance differs across the disorder types.

### 3.4. Predictive Performance of Disorder Predictors Differs across Different Classes of IDPs

We studied the differences in the predictive performance of the top ten disorder predictors across the different types of disorder. We note that the approach in Section 2.2 catalogs IDRs in the way that some of them could belong to multiple categories, e.g., long IDRs that are binding. However, the assessment of disorder predictions must be done at the protein level, and thus we adapt the IDR-based approach to categorize IDPs. Correspondingly, we group IDPs into the following six classes: (1) fully disordered proteins (disorder content ≥ 0.8); (2) low disorder content proteins with short IDRs (disorder content ≤ 0.3 and IDRs ≥ 10 and <15 AAs long); (3) low disorder content proteins with binding long IDRs (disorder content ≤ 0.3 and binding IDRs > 15 AAs long); (4) low disorder content proteins with non-binding long IDRs (disorder content ≤ 0.3 and non-binding IDRs > 15 AAs long); (5) high disorder content proteins with binding IDRs (0.3 < disorder content < 0.8 and binding IDRs); and (6) high disorder content proteins with non-binding IDRs (0.3 < disorder content < 0.8 and non-binding IDRs). Table 2 provides the AUC values of the leading disorder predictors for the entire CAID dataset and each of the six classes of IDPs.

First, we analyze whether these results align with the analysis of the compositional bias from Figure 2. The lowest KCC values when compared against the CAID disorder are for the fully disordered proteins and the short IDRs (Figure 2). These two disorder types should be the hardest to predict since they have the most dissimilar bias when compared to the generic CAID disorder. Correspondingly, using Table 2, we find that the average AUC over the ten predictors for the fully disordered proteins (class 1) is 0.60, and for the proteins with short IDRs (class 2) is 0.69. In contrast, the long IDRs and binding IDRs have high values of KCC and thus they should be easier to predict based on the high similarity of their compositional bias (Figure 2). As expected, based on Table 2, the average AUC among the ten predictors for the IDPs with long IDRs (classes 3 and 4) is 0.73 and for the IDPs with binding IDRs (classes 3 and 5) is 0.71. This confirms that the compositional bias influences the predictive performance of the current methods.

Second, we use Table 2 to analyze the performance of disorder predictors across different classes of IDPs. The flDPnn method, which secures the best overall result on the entire dataset, obtains the highest AUC for the three classes of IDPs that have low disorder content. However, flDPnn is outmatched by other predictors for the other three categories of IDPs. RawMSA secures the highest AUCs for the fully disordered proteins and IDPs with a high disorder content that include binding IDRs. In particular, for the fully disordered proteins, RawMSA outperforms the second-best flDPlr by a wide margin (0.801 vs. 0.687). Finally, SPOT-Disorder1 has the best AUC = 0.866 for the IDPs with a high disorder content that exclude binding IDRs. This means that there is no universally best disorder predictor that outperforms the other methods across all classes of disorder. Moreover, this suggests that matching predictors to the disorder classes where they perform favorably should lead to substantial improvements in predictive performance when compared to using a single tool.

### 3.5. Matching Disorder Predictors to Specific Classes of IDPs Substantially Improves Predictive Performance

Using the results from Table 2, we select the best method for each IDP class and combine their predictions together, resulting in a meta-predictor. To be more specific, we normalize the scores produced by these methods using the min–max approach and use RawMSA to predict the fully disordered IDPs (class 1), flDPnn for IDPs with the low disorder content (classes 2, 3 and 4), RawMSA for the high disorder content IDPs with binding IDRs (class 5) and SPOT-Disorder1 for the high disorder content IDPs with non-binding IDRs (class 6). We quantify the predictive performance using a comprehensive collection of metrics that were utilized in the CAID assessment [49], including AUC, the area under the precision–recall curve (AUPR), F1 and the Matthews correlation coefficient (MCC). We also assessed the statistical significance of differences in the predictive performance between the meta-method and each of the top ten disorder predictors using the procedure described in Section 2.3.

Table 3 compares the predictive quality of the top ten disorder predictors and the meta-method. The AUC of the meta-method reaches 0.855 and is statistically significantly higher than the AUCs of all other predictors, including the best individual predictor, flDPnn, which secures AUC = 0.814 (*p*-value < 0.05). Similarly, the meta-method secures AUPR = 0.605, MCC = 0.474 and F1 = 0.560 when compared to the second highest AUPR = 0.479 for AUCpreD, the second highest MCC = 0. 358 and F1 = 0.462 for flDPnn; these differences are statistically significant (*p*-value < 0.05). We note large margins of improvements at approximately 0.04 for AUC and 0.13 for AUPR, which demonstrate that combining methods that best fit a given disorder class leads to substantial gains in the predictive quality. However, we emphasize that the meta-approach that we describe here is impractical since the selection of the appropriate predictor depends on prior knowledge of the disorder class.

## 4. Conclusions

IDRs are characterized by a sequence bias that is distinct from the sequences of structured regions. This bias at the amino acid level is captured by the TOP-IDP scale [15]. We find that this scale is largely consistent with the bias that we compute using annotations of disorder from the CAID experiment. We find that the six most disorder-promoting AAs include P, E, S, K, D and G while the most order-promoting residues are W, F, Y, I, L and C. Moreover, IDRs carry out many diverse cellular functions and differ in size and placement in the protein sequence. This diversity leads to variations in the underlying sequence biases. Prior studies demonstrate a strong amino acid composition bias of IDRs [1,4,9,10,11,12,13,14], including works that identify differences in this bias between short and long IDRs [76,77]. We analyze the compositional bias of IDRs at a finer granularity by considering four classes of disorder: fully disordered proteins, short IDRs, long IDRs and disordered binding regions. Our empirical analysis finds three distinct types of biases: one that underlies the fully disordered proteins, one that is shared by the long and binding IDRs and the third for the short IDRs.

Motivated by the large number and diversity of the sequence-based disorder predictors [30,36,37,41,42], we utilize the recently released CAID results to investigate the compositional bias of the putative disorder generated by the top performing predictors. We found that the compositional bias of the putative disorder is very similar to the bias of the native disorder. Moreover, the accuracy of the predictions across different methods is highly correlated with the level of correctness of their corresponding compositional biases. This suggests that the accurate compositional bias of the putative disorder is an important characteristic for modern disorder predictors, which to a large degree explains/determines their predictive performance.

We tie these two investigations together by quantifying and studying variations in the performance of disorder predictors across different classes of disorder. We find that an average predictive quality measured across the considered disorder predictors is relatively low for the disorder classes that have compositional bias that is the most different from the “generic” disorder bias, which include the fully disordered proteins and the short IDRs. Moreover, disorder predictions are more accurate for long IDRs and binding IDRs for which compositional bias is the most correlated with the “generic” disorder bias. This further supports the importance of compositional bias to the predictive performance of the current methods.

We also empirically find that different disorder predictors perform best across different classes of disorder. This suggests that no single predictor can claim to be universally the best. Moreover, we discover that the predictive performance of a meta-method that utilizes the best predictors for their matching disorder classes is significantly better than the performance of the best current predictors. While such a meta-method is impractical, as it requires a priori knowledge of the disorder class, this result motivates the development of new designs of disorder predictors where multiple models that target predictions of specific disorder classes are combined together. Similar methods were designed in the past where models that aim to make predictions of short and long IDRs are combined using machine learning algorithms [102,103,104,105,106]. These methods were rather successful in prior community assessments, with VSL2 being ranked among the most accurate methods in CASP7 [46] and MFDp ranking third in CASP10 [48]. Our study advocates further research in this vein that would consider a finer categorization of the disorder classes. Another alternative is to build a meta-model by selecting a disorder predictor based on intrinsic characteristics of the predictions (e.g., use different predictors for proteins where the putative disorder content is high vs. low or when putative binding IDRs are predicted) or the underlying protein sequence. One example of the former approach is the DISOselect tool [107]. DISOselect recommends the best-performing disorder predictor based on a tree regressor model that relies on selected sequence-derived properties, such as the estimated propensity for secondary structures, hydrophobicity and charge. However, the use of DISOselect is limited to 12 disorder predictors that exclude some of the most recent and accurate tools, for example AUCpreD, DisoMine, flDPlr, flDPnn, Predisorder, RawMSA and SPOT-Disorder2.

## Figures and Tables

**Table 1:** Summary of IDPs and IDR data in the CAID dataset.

Protein Set	No. Proteins	No. IDRs	No. Disordered Residues	Median IDR Length	Average IDR Length
**Complete dataset**	652	838	54,820	34	65.5
**Fully disordered proteins**	56	57	9208	132	157.6
**Short IDRs**	124	148	1810	12	12.2
**Long IDRs**	71	77	14,935	139	193.9
**Disordered binding regions**	232	256	21,389	54	83.6

**Table 2:** Predictive performance measured with AUC for the top ten disorder predictors on the CAID dataset and for the six types of IDPs from the CAID dataset. The bold font identifies the methods that secure the highest AUC for a given collection of IDRs. Predictors are sorted alphabetically. We computed the results in the first row and they reproduce the original results from the CAID article [49].

Dataset	AUCpreD	AUCpreD-np	DisoMine	flDPlr	flDPnn	Predisorder	RawMSA	SPOT-Disorder1	SPOT-Disorder2	SPOT-Disorder-Single
CAID dataset	0.757	0.751	0.765	0.793	**0.814**	0.747	0.780	0.744	0.760	0.757
Fully disordered proteins	0.475	0.505	0.612	0.687	0.666	0.636	**0.801**	0.502	0.547	0.621
Low disorder content with short IDRs	0.715	0.698	0.654	0.703	**0.736**	0.708	0.651	0.675	0.687	0.678
Low disorder content with binding long IDRs	0.669	0.664	0.649	0.723	**0.751**	0.661	0.711	0.635	0.693	0.658
Low disordered content with non-binding long IDRs	0.801	0.785	0.747	0.802	**0.816**	0.778	0.806	0.771	0.779	0.779
High disordered content with binding IDRs	0.732	0.718	0.686	0.732	0.731	0.735	**0.760**	0.716	0.732	0.726
High disordered content with non-binding IDRs	0.824	0.815	0.799	0.726	0.737	0.816	0.811	**0.866**	0.808	0.824

**Table 3:** Predictive performance measured with AUC, AUPR, MCC and F1 for the top ten disorder predictors and the meta-method on the CAID dataset. The bold font identifies the highest value for a given metric. “*” means that the difference between the best-performing meta-method and a given disorder predictor is statistically significant at *p*-value of 0.05. Methods are sorted by their AUC value.

Predictors	AUC	AUPR	MCC	F1
Meta-method that selects the best predictor for each disorder class	**0.855**	**0.605**	**0.474**	**0.560**
flDPnn	0.814 *	0.475 *	0.358 *	0.462 *
flDPlr	0.793 *	0.422 *	0.323 *	0.433 *
RawMSA	0.780 *	0.414 *	0.288 *	0.404 *
DisoMine	0.765 *	0.388 *	0.244 *	0.367 *
SPOT-Disorder2	0.760 *	0.340 *	0.200 *	0.351 *
AUCpred	0.757 *	0.479 *	0.258 *	0.399 *
SPOT-Disorder-Single	0.757 *	0.318 *	0.221 *	0.348 *
AUCpred-np	0.751 *	0.428 *	0.226 *	0.349 *
Predisorder	0.747 *	0.325 *	0.227 *	0.359 *
SPOT-Disorder1	0.744 *	0.268 *	0.143 *	0.284 *

## Data Availability

Not applicable.
